# MEDICAID FINANCING AND SEGREGATION OF DUALS IN ASSISTED LIVING

**DOI:** 10.1093/geroni/igac059.1653

**Published:** 2022-12-20

**Authors:** Kali Thomas, Portia Cornell, Cassandra Hua, Momotazur Rahman

**Affiliations:** Brown University, Providence, Rhode Island, United States; Providence VA Medical Center / Brown University, Providence, Rhode Island, United States; Brown University School of Public Health, Providence, Rhode Island, United States; Brown University, Providence, Rhode Island, United States

## Abstract

We used 2018 Medicare enrollment data, a national directory of licensed ALs, and Medicaid state policies. We identified a cohort of 474,661 AL residents and a comparison cohort of 58,911,266 community-residing individuals. We compared the distribution of duals across ALs to the distribution of community-dwelling duals across ZIP codes by taking the ratio of AL Gini index for each state over the community Gini index for each state (the “Gini ratio” or GR). On average, states with both waivers and state plans covering services in AL had the lowest Gini ratio (less segregated than community; GR=0.87;) States with no Medicaid financing for AL had the highest Gini ratio (more segregated than community, GR=1.16). Medicaid coverage for home and community-based services in AL is associated with increased access to AL for duals.

